# Elevated levels of thymidine kinase 1 peptide in serum from patients with breast cancer

**DOI:** 10.1080/03009730802688835

**Published:** 2009-04-24

**Authors:** Lena Carlsson, Anders Larsson, Henrik Lindman

**Affiliations:** ^1^Department of Medical Sciences, Clinical Chemistry, University HospitalUppsalaSweden; ^2^Department of Oncology, University HospitalUppsalaSweden

**Keywords:** Antibodies, breast cancer, immunological sandwich assay, thymidine kinase

## Abstract

**Objectives:**

Thymidine kinase (TK) has an important role in DNA synthesis and is thus related to cell proliferation and turn-over. Traditionally, TK has been measured by enzymatic activity or radioimmunoassays. These assays are difficult to adapt to random access instruments. The aim of this study was to evaluate a new immunological sandwich assay for detection of TK peptides in serum from breast cancer patients.

**Material and methods:**

Serum samples were collected from patients with breast cancer and stored frozen at −70°C. The samples were collected after surgery, after metastatic tumor recurrence and after chemotherapy due to tumour recurrence. Patients’ serum samples were analysed by the TK enzyme-linked immunosorbent assay (ELISA).

**Results:**

In receiver operating characteristics (ROC) analyses of TK1 for diagnosis of breast cancer, the area under the curve (AUC) collected four weeks after surgery was 0.56 (95% CI 0.47–0.65), for samples collected postsurgically after tumour recurrence 0.73 (95% CI 0.65–0.80), and after chemotherapy 0.64 (95% CI 0.56–0.72).

**Conclusions:**

This study indicates that the tumour proliferation marker TK has a potential as a serum marker in breast cancer. Further studies are warranted to verify this observation.

## Introduction

Thymidine kinase (TK) (EC.2.7.1.21) is an enzyme in the pyrimidine salvage pathway and catalyses the phosphorylation of thymidine to thymidine monophosphate (1). There are two forms of TK: a cytosolic (thymidine kinase 1 (TK1)) and a mitochondrial one (TK2) (2). The level of TK1 is very low in non-proliferating cells but increases dramatically at late G1 to late S-phase/early G2 phase during the cell cycle in proliferating cells and tumour cells. This makes TK1 an interesting marker for cell proliferation and tumour growth. In patients with malignancies more than 95% of the TK1 activity in serum is derived from the malignant cells (3). Thus, serum TK1 should be a good marker for tumour cell proliferation. Serum TK1 activity has been used to monitor the extent of tumour metastasis and prognosis in patients with acute leukaemia, chronic leukaemia, Hodgkin's and non-Hodgkin's lymphoma, bladder carcinoma, and cervical carcinoma (4–10). The most widely used TK1 assays are radioimmunoassay techniques, discouraging their routine clinical use. It is therefore important to find non-radioactive alternatives to these assays. Both monoclonal and polyclonal antibodies are available against TK1. One of these antibodies is a chicken anti-TK1 polyclonal antibody raised against a 31-amino acid synthetic peptide (GQPAG PDNKE NCPVP GKPGE AVAAR KLFAPQ, residues 194–225) corresponding to the C-terminal part of human TK1 (3). This antibody has been used to develop a sandwich enzyme-linked immunosorbent assay (ELISA) for TK1.

Breast cancer incidence has approximately doubled compared with 1960. The number of new breast cancer cases in Sweden in the year 2001 was 6,569, resulting in an age-adjusted incidence of 138 per 100,000 women (11). The number of deaths from breast cancer was 1,487, giving a mortality rate of 30 per 100,000. Breast cancer represented 29% of all new cancers in women, and for a Swedish woman the lifetime risk of developing breast cancer is now above 12% (11). Most new breast cancers are detected in women aged 55–59 years. The incidence of and mortality from breast cancer is markedly higher in Western countries than in Asian or African countries (12). The average annual age-standardized breast cancer rate increase in Sweden has been 1.8% during the last decade, but the mortality figures have slowly decreased and are, despite the high incidence, one of the lowest in Europe (13). The annually increasing cure rate, since the late 1980s also observed in the United States and the United Kingdom, could have many explanations, but earlier diagnosis and more efficient adjuvant therapies are corner-stones that cannot be neglected (14).

A proliferation marker such as TK1 could theoretically be used to distinguish between those cancers that proliferate slowly and more malignant forms that show rapid growth. The present report is the first clinical evaluation of a new sensitive and specific immunoassay (enzyme-linked immunosorbent assay, ELISA) for determination of thymidine kinase 1 in serum from breast cancer patients.

## Material and methods

### Patients’ sera and control sera

Blood samples were collected from patients with breast cancer treated at the Department of Oncology, Uppsala University Hospital. Twenty-four samples were collected four weeks after surgery, 39 samples were collected after postsurgical tumour recurrence, and 41 samples were collected after chemotherapy. A group of 100 healthy blood donors from Uppsala University Hospital Blood Bank were used as controls. This study was approved by the local Ethics Committees.

### TK1 enzyme-linked immunosorbent assay (ELISA)

Samples were analysed using an ELISA kit for thymidine kinase (TK1) (Arocell, Uppsala, Sweden). Briefly, the microtitre plates had been coated with affinity-purified polyclonal chicken antibodies specific for TK1 peptide, and the first step was to add standards and samples to the wells. During the subsequent incubation period TK1 present in standards and samples was bound to the immobilized antibody.

The plate was washed four times, and a biotinylated polyclonal antibody specific for TK1 was added. After incubation and four washes, enzyme-labelled streptavidin was pipetted into the wells, and, following an incubation step and four washes, a substrate solution was added and colour developed in proportion to the amount of TK1 bound. The colour development was subsequently stopped, and the intensity of colour was measured by spectrophotometry. Calculation of results was performed according to manufacturer's recommendations, and TK1 concentration was expressed in U/L.

### Statistical analyses

Receiver operating characteristics (ROC) curves were plotted, and areas under the ROC curves were compared for specificity and sensitivity in the three breast cancer groups. Sensitivity (the ratio of patients with breast cancer who were positive for the variable) and specificity (the ratio of patients without breast cancer who were negative for the variable), negative predictive value (probability that the disease is not present when the test is negative), and positive likelihood ratio (ratio between the probability of a positive test result given the presence of the disease and the probability of a positive test result given the absence of the disease) were also calculated. These analyses were performed on MedCalc Software (Mariakerke, Belgium). Statistical significance was considered at *P <* 0.05.

## Results

### Clinicopathological characteristics of the patients

Median value for the samples collected four weeks after surgery was 7.05 U/L (range 0.8–130.8 U/L), the median value for samples collected after postsurgical tumour recurrence was 13.7 U/L (range 0.8–170.1 U/L), and median value for samples collected after chemotherapy was 9.1 U/L (range 0.4–199.8 U/L).

The mean value for the controls was 5.78 U/L (range 1.5–159.4 U/L).

### Thymidine kinase 1 ELISA titres

**Figure 1.**
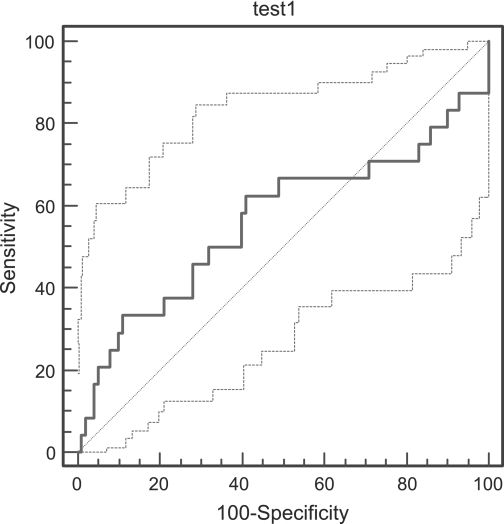
Receiver operating characteristics (ROC) curves for thymidine kinase 1 (TK1) analysed in serum samples from patients with breast cancer (*n*=24) collected four weeks after surgery in comparison with healthy controls (*n*=100). Area under the curve (AUC) = 0.56, 95% confidence interval (CI) 0.47–0.65, *P*=0.34.

Twenty-four samples were collected four weeks after surgery. The accuracy of the TK1 ELISA to detect breast cancer prior to surgery according to receiver operating characteristics is given in Figure 1. The area under the curve (AUC) for this patient group was 0.56 (95% CI 0.47–0.65, *P =* 0.34). The sensitivity of the assay was 33.3% and the specificity 89% using a cut-off of 12.3.

**Figure 2.**
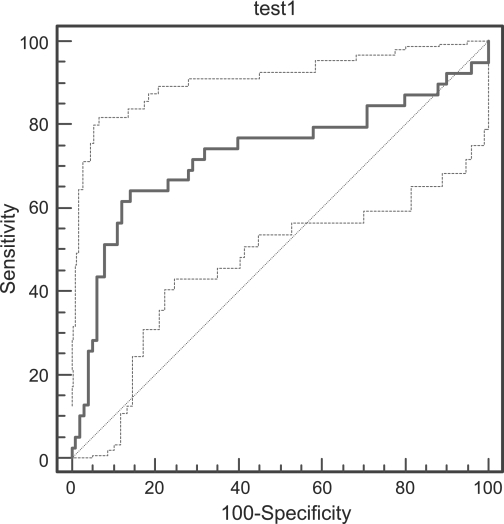
Receiver operating characteristics (ROC) curves for thymidine kinase 1 (TK1) analysed in serum samples from patients with breast cancer (*n*=39) collected after metastatic tumour recurrence after surgery in comparison with healthy controls (*n*=100). Area under the curve (AUC) = 0.73, 95% confidence interval (CI) 0.65–0.80, *P*<0.0001.

Thirty-nine samples were collected upon postsurgical tumour recurrence. The accuracy of the TK1 ELISA to detect breast cancer in this patient group according to receiver operating characteristics is given in Figure 2. The AUC for this patient group was 0.73 (95% CI 0.65–0.80, *P <* 0.0001). The sensitivity of the assay in this patient group was 64% and the specificity 86% using a cut-off of 11.0.

**Figure 3.**
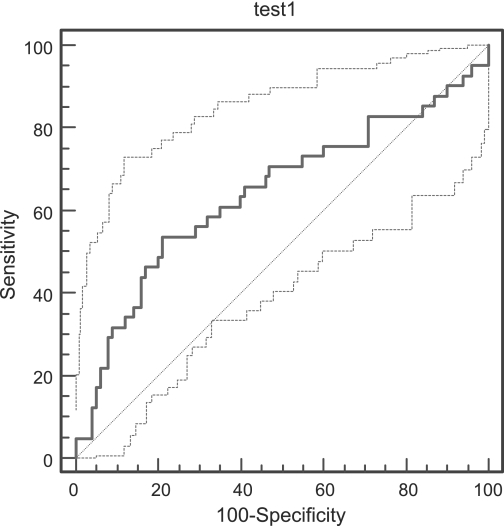
Receiver operating characteristics (ROC) curves for thymidine kinase 1 (TK1) analysed in serum samples from patients with breast cancer (*n*=41) collected after chemotherapy in comparison with healthy controls (*n*=100). AUC = 0.64, 95% CI 0.56–0.72, *P =* 0.0076.

Forty-one samples were collected after chemotherapy. The AUC after chemotherapy was 0.64 (95% CI 0.56–0.72, *P =* 0.008) (Figure 3). The sensitivity of the assay in this patient group was 53.7% and the specificity 79.0% using a cut-off of 8.9.

## Discussion

TK1 activity is low or absent in resting cells, starts to occur in late G1 cells, increases in S- phase, and disappears during mitosis. The cell cycle regulation makes TK1 a highly interesting marker for cell proliferation and as a marker for tumour growth. Also, TK1-deficient mice have been shown to have increased mutation rates (15). The role of TK1 in tumour growth has led to the use of TK1 as a cancer serum marker.

The present study shows that TK1 can be a potential tumour marker in breast cancer patients. This result could reasonably be explained by the fact that the level and activity of TK1 are highly dependent on the growth state and cell cycle phase and that the transcription of the TK1 gene is strongly induced in response to growth stimulation in the S-phase (16–19).

The assay used in the present study is a non-radioactive immunological alternative to the traditional TK1 radioimmunoassay. The assay could also be adopted to fit the format of high throughput immunoanalysers currently used in clinical laboratories. This would facilitate the use of TK1 as a tumour marker. The anti-TK1 Immunoglobulin Y (IgY) antibody used in the ELISA has previously been characterized (3). Antibodies derived from egg-yolk offer interesting advantages over mammalian antibodies in several aspects and may sometimes be a more suitable choice in designing solid-phase immunometric assays than mammalian antibodies. Chicken antibodies do not activate the human complement system which is a well known source of interference in sandwich immunoassays (20). Capture antibodies bound to a solid surface are potent complement activators, and the activated complement components will react with the assay antibodies thus partly blocking the antigen-binding sites (21). Another advantage is that rheumatoid factors (RF) do not bind to IgY. RF is a major source of interference in many immunoassays, reacting with the Fc portion of mammalian IgG (22). The disease usually associated with RF is rheumatoid arthritis, but RF is also present in blood samples from patients with many other diseases and also healthy individuals (6,7). Most immunoassays use mammalian polyclonal or monoclonal antibodies, which are subjected to RF binding, thus potentially giving false positive results. As RF is not able to bind to IgY, chicken antibodies can be useful in assays (e.g. nephelometry, turbidimetry, or ELISA) where RF could interfere (22). Another interfering factor is human anti-mouse IgG antibody (HAMA). An increasing number of patients are *in vivo* treated with monoclonal mouse antibodies, and this often provokes an antibody response in the patient resulting in HAMA production. Chicken antibodies do not react with HAMA, so they can be used to eliminate such interference (23,24). Thus, chicken antibodies should theoretically have advantages over mammalian antibodies in immunoassays especially for tumour marker assays as mouse monoclonal antibodies are used for tumour treatment and this treatment increases the prevalence of HAMA (24).

In conclusion, the current study has demonstrated that the serum concentration of TK1 is increased in patients with breast cancer. These findings could provide the basis for future studies of the clinical utility of the assay.
